# Classification of Breast Cancer Using Transfer Learning and Advanced Al-Biruni Earth Radius Optimization

**DOI:** 10.3390/biomimetics8030270

**Published:** 2023-06-26

**Authors:** Amel Ali Alhussan, Abdelaziz A. Abdelhamid, S. K. Towfek, Abdelhameed Ibrahim, Laith Abualigah, Nima Khodadadi, Doaa Sami Khafaga, Shaha Al-Otaibi, Ayman Em Ahmed

**Affiliations:** 1 Department of Computer Sciences, College of Computer and Information Sciences, Princess Nourah Bint Abdulrahman University, P.O. Box 84428, Riyadh 11671, Saudi Arabia; 2 Department of Computer Science, College of Computing and Information Technology, Shaqra University, Shaqra 11961, Saudi Arabia; 3 Department of Computer Science, Faculty of Computer and Information Sciences, Ain Shams University, Cairo 11566, Egypt; 4 Computer Science and Intelligent Systems Research Center, Blacksburg, VA 24060, USA; 5 Department of Communications and Electronics, Delta Higher Institute of Engineering and Technology, Mansoura 35111, Egypt; 6 Computer Engineering and Control Systems Department, Faculty of Engineering, Mansoura University, Mansoura 35516, Egypt; 7 Computer Science Department, Prince Hussein Bin Abdullah Faculty for Information Technology, Al al-Bayt University, Mafraq 25113, Jordan; 8 Hourani Center for Applied Scientific Research, Al-Ahliyya Amman University, Amman 19328, Jordan; 9 MEU Research Unit, Middle East University, Amman 11831, Jordan; 10 School of Computer Sciences, Universiti Sains Malaysia, Pulau Pinang 11800, Malaysia; 11 Department of Civil and Architectural Engineering, University of Miami, Coral Gables, FL 33146, USA; 12 Department of Information Systems, College of Computer and Information Sciences, Princess Nourah Bint Abdulrahman University, P.O. Box 84428, Riyadh 11671, Saudi Arabia; 13 Faculty of Engineering, King Salman International University, El-Tor 8701301, Egypt

**Keywords:** biological mechanism, cancer detection, Al-Biruni Earth radius optimization algorithm, machine learning

## Abstract

Breast cancer is one of the most common cancers in women, with an estimated 287,850 new cases identified in 2022. There were 43,250 female deaths attributed to this malignancy. The high death rate associated with this type of cancer can be reduced with early detection. Nonetheless, a skilled professional is always necessary to manually diagnose this malignancy from mammography images. Many researchers have proposed several approaches based on artificial intelligence. However, they still face several obstacles, such as overlapping cancerous and noncancerous regions, extracting irrelevant features, and inadequate training models. In this paper, we developed a novel computationally automated biological mechanism for categorizing breast cancer. Using a new optimization approach based on the Advanced Al-Biruni Earth Radius (ABER) optimization algorithm, a boosting to the classification of breast cancer cases is realized. The stages of the proposed framework include data augmentation, feature extraction using AlexNet based on transfer learning, and optimized classification using a convolutional neural network (CNN). Using transfer learning and optimized CNN for classification improved the accuracy when the results are compared to recent approaches. Two publicly available datasets are utilized to evaluate the proposed framework, and the average classification accuracy is 97.95%. To ensure the statistical significance and difference between the proposed methodology, additional tests are conducted, such as analysis of variance (ANOVA) and Wilcoxon, in addition to evaluating various statistical analysis metrics. The results of these tests emphasized the effectiveness and statistical difference of the proposed methodology compared to current methods.

## 1. Introduction

Cancer is a global health problem. Among female cancers, breast cancer is by far the most common [1]. However, 42 percent of NHS trusts say they cannot assign individuals because they do not have enough staff, with many citing a lack of breast cancer specialists. It is the fundamental reason breast cancer has a dismal survival rate worldwide [2]. Breast cancer specialists are in limited supply, which will delay diagnosis, increase resistance to effective screening and treatment, and create inequalities in access to care [3]. The goal of developing methods for detecting breast cancer was to identify anomalies and classify the disease more accurately. This practice aids in detecting breast cancer [4]. Death rates can be reduced with early detection using screening mammography; however, this is challenging due to the small size of potential nodules concerning the entire breast [5]. Breast cancer has a higher chance of being cured (about 90%) than other cancer types. Cancer patients often go undiagnosed until they experience severe symptoms [6]. The patients’ ages affect the mortality and incidence rates of breast cancer. Breast cancer was typically diagnosed in patients aged 62 between 2010 and 2014 [7].

With an estimated 90,000 new cases annually and a reported 40,000 deaths due to the disease, Pakistan has Asia’s highest breast cancer mortality rate [8,9]. Survival rates for certain cancers vary depending on their detection stages [10]. Those who are predicted to live beyond a certain point after receiving a diagnosis and continue to function normally are included in the survival rate. Mammography is the most reliable technology for identifying breast cancer due to its capabilities and inexpensive cost to satisfy medical requirements. The study of mammograms is the major approach doctors use to make a diagnosis. However, it can be affected by bias and fatigue. Mammography, unfortunately, has a relatively low detection rate. Depending on the kind of the lesion, the breast density, and the patient’s age, it can yield a false-negative result rate of anywhere from 5% to 30% [11]. Mammography uses low-dose radiography because it allows us to see the breast’s internal structure.

To diagnose breast cancer, machine learning algorithms are trained to look for specific patterns and associations in data that are linked to the biological mechanisms through which cancer develops. The aberrant multiplication and proliferation of breast cells are central to the basic mechanisms behind breast cancer, which can have multiple underlying causes, including heredity, lifestyle, and the external environment. These processes can lead to the development of breast abnormalities such as lumps, masses, or cysts, which can be discovered using mammography, ultrasound, or magnetic resonance imaging. These imaging data can be fed into a machine-learning algorithm and trained to look for abnormalities or patterns that are indicative of breast cancer. For breast cancer, machine learning algorithms can be trained to recognize telltale signs such as masses and microcalcifications [12,13]. Imaging data, patient history, and molecular biomarkers are just some data sources that can be analyzed with machine learning algorithms to enhance breast cancer detection. Machine-learning algorithms can improve the accuracy and timeliness of breast cancer diagnostics by merging data from numerous sources to detect tiny changes in breast tissue that may indicate the presence of cancer. Breast cancer risk factors include genetics, lifestyle choices, and other factors; these can all be modeled using machine learning algorithms to create prediction models of an individual’s likelihood of developing breast cancer. These models can be used to inform screening and preventative strategies, which in turn can help lower breast cancer rates. To a large extent, the biological mechanisms of cancer development are intertwined with breast cancer detection using machine learning, as these algorithms are trained to recognize patterns and abnormalities in breast tissue that are linked to the development of cancer [14,15,16].

CNN recently demonstrated promising performance in detecting and categorizing tumors in medical images. Deep learning models’ performance is typically proportional to the size of the datasets used for training. In contrast to the deep learning-based strategies, the traditional methods performed poorly on complex nature datasets. Deep learning employs the concept of CNN to perform breast cancer classification [17,18,19]. Convolutional, pooling, activation, and fully linked layers are some types of layers (hidden layers) seen in a CNN model. Softmax is the classifier used in the final layer of a convolutional neural network model. The use of deep learning enables automated artificial intelligence approaches in medical imaging. Researchers have introduced several deep learning-based architectures to detect and categorize infectious diseases. While several deep learning methods have been established to aid in the classification and diagnosis of breast cancer, researchers have encountered obstacles such as imbalanced datasets, noisy imaging data, and the downsampling of critical features [20]. The team zeroed in on the problem of teaching deep models through transfer learning. One use of transfer learning is to apply a model that has already been trained to a new problem or scenario [21,22]. While hyperparameters such as learning rate, mini-batch size, and others have been used successfully in training, setting their values by hand is tedious and error-prone when dealing with breast cancer. The authors of described an improved hyperparameter-based deep-learning system for breast cancer classification [23]. Extraction of deep features from the fully connected layer followed training; nevertheless, it was shown through analysis that numerous features were redundant, which negatively impacted breast cancer classification [23]. An improved method of classifying breast cancer using deep learning was recently presented by the authors of [24]. The authors of [25] proposed dialectical feature selection to improve breast cancer classification; however, these methods run into the issue of stopping after the ideal values have been retrieved.

Due to its many benefits over alternative modalities, the mammogram has become the preferred modality for screening for breast cancer [26]. First, mammography has been the subject of much research and is useful in identifying breast cancer at an early stage. When used with a clinical breast exam, it can detect small cancers or microcalcifications that the naked eye could miss. Successful treatment results can be improved by prompt action made possible by this early identification. Second, mammograms produce highly detailed pictures of breast tissue, letting radiologists see any irregularities very plainly. This screening method is safe and well-accepted because of the low-dose X-rays used in mammography. As a bonus, mammography can even spot breast cancer in people with thick breast tissue. Breasts often have dense tissue, which might obscure cancers on conventional imaging techniques such as ultrasonography. Mammography is useful for screening women with a wide variety of breast densities because it can successfully penetrate thick tissue. The widespread accessibility and well-established infrastructure of mammography are additional benefits. Most medical facilities, clinics, and screening centers can access mammography equipment. Because of this, many women will be able to get screened regularly, which will help with identification and treatment early on. Compared to other screening methods, mammography also has a low cost. It strikes a good compromise between price and accuracy in establishing a diagnosis, making it a viable option for widespread breast cancer screening programs. Mammography has been widely adopted as the standard screening method for breast cancer because of its efficacy in detecting cancer at an early stage, its high-resolution imaging capabilities, its ability to identify tumors even in thick breast tissue, its widespread availability, and cost-effectiveness. These benefits work together to make breast cancer treatment more effective and decrease patient mortality [27].

### 1.1. Main Contributions of This Work

In this paper, we proposed a new framework that uses deep learning to aggregate the best possible features from both the original and upgraded mammography images. The following is a list of the main contributions achieved throughout this work:

- Employing transfer learning for feature extraction using the pretrained AlexNet deep network.
- Developing a new optimization algorithm based on improving the behavior of the Al-Biruni Earth Radius (BER) optimization algorithm. The new algorithm is referred to as Advanced BER (ABER).
- Optimizing the structure and training parameters of the classification CNN for boosting its performance.
- Two datasets are employed to prove the effectiveness and generalization of the proposed approach.
- Studying the statistical difference of the proposed methodology using ANOVA and Wilcoxon signed ranks tests.
- Applying statistical analysis to show the stability of the proposed methodology in classifying breast cancer cases.

The main motivation for using the BER optimization algorithm is its efficiency in exploring the search space for the best solution. On the other hand, the motivation for using AlexNet is that its performance is better than the other deep networks, such as GoogleNet and VGG. Therefore AlexNet is adopted for feature extraction. In addition, CNN is used for the classification of breast cancer. The BER optimization algorithm is used to optimize its parameters to achieve the best performance of the CNN.

### 1.2. The Structure of This Work

The structure of this work proceeds as follows. The literature review is presented in Section 2. The details of the proposed methodology are presented and discussed in Section 3. The achieved results of the conducted experiments and comparisons are then discussed in Section 4. Finally, the conclusions are future perspectives are presented in Section 5.

## 2. Literature Review

Around 1.7 million women were diagnosed with cancer in 2012. Breast cancer is the most frequent type of cancer worldwide. Risk factors for breast cancer include age, family history, and previous health problems [4]. Women account for the lion’s share of cancer deaths; annually, an estimated 2.1 million people are diagnosed with breast cancer. Recent research estimates that 627 thousand women lost their lives to cancer in 2018, accounting for fifteen percent of all cancer deaths in women [5]. It is usual practice to use a deep learning-based model for breast cancer diagnosis and classification when using computer visualization. Clinicians face difficulties in making a cancer diagnosis from mammography scans due to the complexity of early breast cancer and the fading of images. That is why it is so important to enhance a doctor’s detection efficiency with the help of deep learning algorithms used in the CAD system [28,29,30,31].

To categorize breast cancer, the authors of [4] proposed a convolutional neural network (CNN) based framework for analyzing mammography images. In the beginning, preprocessing was carried out so the mammography images could be seen. Then, the deep learning model that was used to extract the features was trained using the preprocessed images. Softmax, a CNN classifier, was then used to categorize the last layer’s retrieved features. The preferred model enhanced the introduced framework’s classification accuracy of mammography images. Accuracy values of 0.8585 and 0.8271 for the proposed framework demonstrate its superiority to those of the state-of-the-art alternatives. The authors of [32] revealed early results for utilizing transfer learning to identify breast abnormalities likely to progress to cancer. After testing numerous deep learning models, they settled on ResNet50 and MobileNet as the best options. Both models achieved the highest accuracy levels (78.4% and 74.3%, respectively). They used several preprocessing methods to enhance the accuracy of the categorization further. Last but not least, in [33], researchers introduced a novel hybrid processing approach that combines principle component analysis (PCA) and logistic regression (LR).

Using a multi-view screening image-processing architecture, the authors of [34] were able to improve diagnostic results. First-order local entropy, a texture-based technique, segmented the tumor patches. Malignancy indicators such as radius and area were derived using the feature extraction findings. Results from applying this strategy indicated that the CC and MLO views were 88% and 80% accurate at detecting breast cancer, respectively. The framework described by the authors in [35] centered on transferable knowledge. Several augmentation methods are employed to increase the total number of mammograms without overfitting and produce accurate findings. Using the enormous mammography images dataset, the authors of [36] proposed a method. A segmentation module is then used to identify breast cancer abnormalities in an image that is properly improved. The Breast Imaging and Reporting and Data System dataset comprised five groups and achieved 92% precision.

Tumor identification with thresholding and CNN methods were the primary focus of the previous research, along with information fusion, hyperparameter value selection by hand, data enhancement, and manual hyperparameter tuning. However, they failed to take key measures that could have increased precision. These processes consist of improving the contrast and then optimizing the retrieved features. The SGD and ADAM optimizers are frequently used to fine-tune the weights of a deep model. A feature optimization method is implemented following the feature extraction stage to combat computational complexity, overfitting, and poor accuracy. Table 1 presents a summary of the related works. This table presented the related works in terms of the presented methodology, the advances, disadvantages, and overall performance. As shown in this table, the low accuracy of most methods represents the research gap addressed through the methodology proposed in this work.

## 3. Proposed Methodology

**Figure 1:**
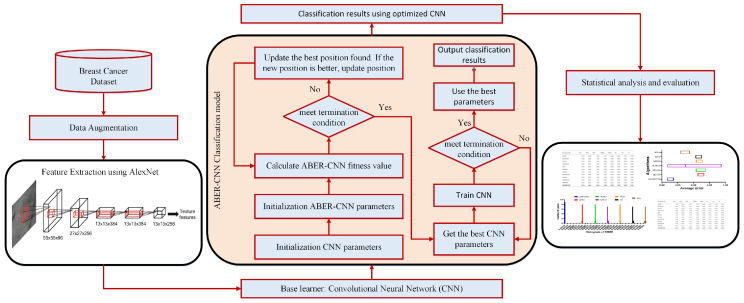
The stages of the proposed methodology.

The proposed framework for mammogram-based breast cancer classification is presented in this section. The steps of the proposed methodology are shown in Figure 1. This figure starts with adopting the breast cancer dataset, followed by data augmentation to enhance these datasets. The next step is feature extraction, in which pre-trained models are employed to realize this step. The pre-trained models include AlexNet, GoogleNet, and VGG. The features extracted from the pre-trained model are then fed to the proposed optimization algorithm to optimize a custom convolutional neural network (CNN) parameter. The proposed optimization algorithm is based on an improved Al-Biruni Earth Radius (BER) optimization algorithm which is denoted by advanced BER (ABER). After optimizing the parameters of the CNN, it is used to classify the test images of the given datasets. The classification results are finally analyzed using several evaluation criteria and statistical methods. The next sections present more details about these steps.

### 3.1. Dataset

The Digital Database for Screening Mammography (DDSM) dataset employed in this research can be accessed at [45]. Dataset-1 denotes this dataset throughout this text. It provides a large database of mammograms, both normal and abnormal. A suggested optimal convolutional neural network (CNN) for classification uses this dataset for training and testing. CNN is a robust deep-learning model developed especially for analyzing and interpreting visual input, making it excellent for mammography classification applications. Accurate categorization of mammograms may be accomplished using this dataset in conjunction with the suggested optimized CNN. The breast pictures are sent into a deep learning network, which then learns complex patterns and characteristics to identify anomalies such as masses, calcifications, and architectural deformities. By applying the suggested optimization strategies to the CNN design, we may boost its performance in terms of overfitting reduction, generalization, and classification precision. Researchers can use this dataset to test how well the improved CNN works. They can use a smaller sample of the data for model training and then verify its accuracy using a larger test set. Together, the proposed optimized CNN and the DDSM mammography dataset provide a robust system for the classification of mammograms. The enhanced CNN, which uses deep learning techniques with the dataset, can improve the accuracy and efficiency with which mammograms are classified, hence facilitating the early identification and diagnosis of breast problems. The number of images in this dataset is 1696 images including benign and malignant cases.

An additional dataset is considered to emphasize the effectiveness of the proposed methodology. This dataset is publicly available on Kaggle [46] and is denoted by Dataset-2 throughout this text. The dataset available at the provided link is a collection of mammograms and breast cancer images. It is a valuable resource for training and evaluating a proposed optimized convolutional neural network (CNN) for classification purposes. By leveraging this dataset, the proposed optimized CNN can be trained to classify breast cancer images and mammograms accurately. Utilizing this dataset in conjunction with the optimized CNN can improve the efficiency and accuracy of a breast cancer diagnosis significantly. The deep learning model can learn intricate patterns and features from the images, enabling it to distinguish between malignant and benign cases. The proposed optimization techniques applied to the CNN architecture can enhance its performance by reducing overfitting, improving generalization, and increasing the overall accuracy of the classification. With this dataset, researchers can thoroughly evaluate the performance of the optimized CNN. The dataset’s diverse range of images and associated metadata allow for a comprehensive evaluation of the proposed optimized CNN across various patient demographics and imaging techniques. The dataset provided, and the proposed optimized CNN presents a promising approach for classifying mammograms and breast cancer images. By harnessing the power of deep learning and leveraging this dataset, the optimized CNN can contribute to accurate and efficient breast cancer diagnosis, and thus improves patient outcomes and better healthcare practices. The number of images in this dataset is 1356 images including benign and malignant cases.

### 3.2. Data Augmentation

**Figure 2:**
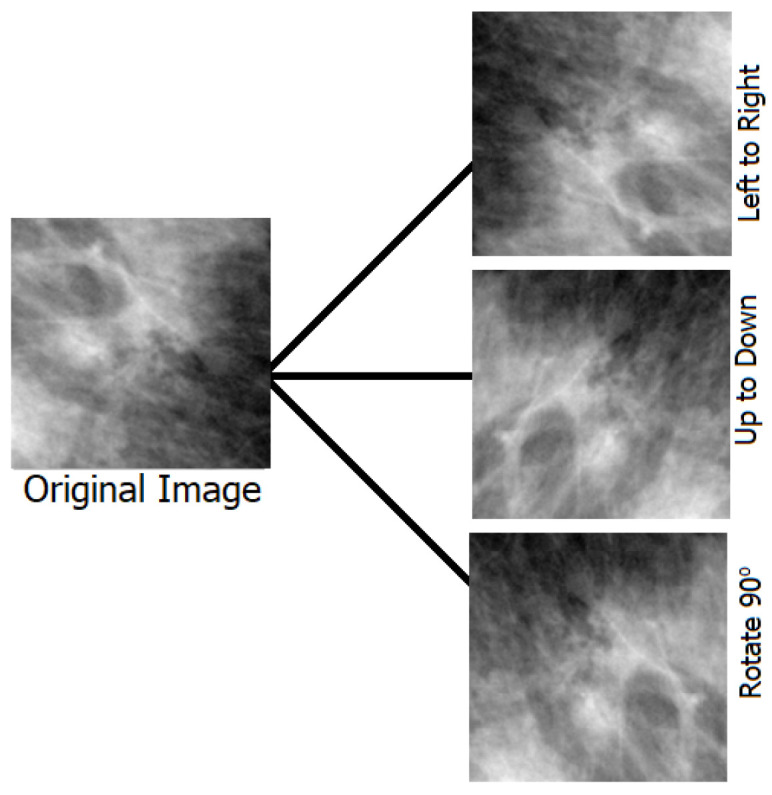
The operations used in the data augmentation process.

Typical machine learning methods, such as those for recognizing shapes, points, colors, and others, benefit from the limited number of image datasets available for training [47,48]. More datasets are constantly needed for developing deep learning models. Overfitting problems are mitigated, and the deep learning model’s robustness is improved through data augmentation, which also increases the size of the dataset. We undertook data augmentation since the publicly accessible datasets for breast cancer are insufficient by rotating each image four times at (0 degrees), (90 degrees), (180 degrees), and (270 degrees), and then flipping the resulting four images from left to right, a total of eight additional shots were generated for each recognized patch. Algorithm 1 presents the steps of the data augmentation process employed in this work to increase the number of images in the dataset. Table 2 presents the number of images in the dataset before and after data augmentation. Samples of the augmentation results are shown in Figure 2.
**Algorithm 1**: The steps of data augmentation1:**while** i = 1 to target augmentation percentage **do**2:   **Step 1:** Input image3:   **Step 2:** Flip right to left4:   **Save** image from step 25:   **Step 3:** Flip-up to down6:   **Save** image from step 37:   **Step 4:** Image rotation to 908:   **Save** image from step 49:**end while**

### 3.3. Feature Extraction

**Figure 3:**
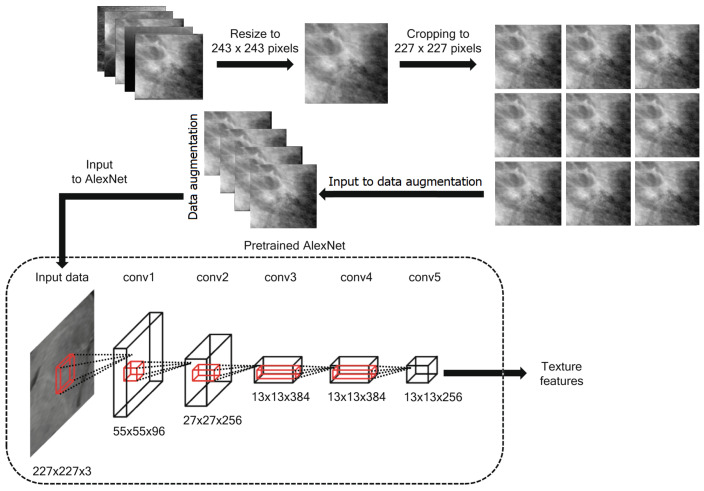
The process of extracting features from the breast cancer images dataset.

During the 2012 ImageNet Large Scale Visual Recognition Challenge (ILSVRC-2012), a new CNN architecture called AlexNet was proposed in [49]. AlexNet is an effective and simple CNN architecture composed of several cascading stages, including fully connected layers, rectified linear unit (ReLU) layers, pooling layers, and convolution layers. Specifically, AlexNet consists of five convolutional layers, three of which are followed by a pooling layer and three fully connected layers. AlexNet uses several pragmatic strategies contributing to its impressive performance, including the dropout regularization technique and ReLU non-linearity layer. The optimization of AlexNet architecture using the stochastic gradient descent (SGD) algorithm is based on back-propagation to optimize the cost function when the convolutional kernels are extracted. The convolutional layers generally apply sliding convolutional kernels to the input feature maps to produce convolved feature maps. The pooling layers aggregate information within a given neighborhood window by performing either a max pooling or an average pooling operation on the convolved feature maps. The ReLU function acts as a half-wave rectifier function, reducing training time and preventing overfitting. Dropout can be seen as a regularization method that randomly sets several hidden neurons or input neurons to zero during training. On the other hand, the dropout regularization technique is commonly used in the fully connected layers of AlexNet architecture to reduce overfitting. Figure 3 shows the steps of the proposed feature extraction method.

The transfer technique and the pre-training procedure [50,51] allow the parameters of a Neural network to be imported from natural imaging datasets. This was partly possible because remote sensing imagery and natural imaging datasets are similar and comparable in terms of their respective categories. Well-trained network parameters are critical for launching the subsequent classification framework, and it makes sense that these parameters may be obtained by training an AlexNet architecture on massive and complicated ImageNet datasets. Therefore, the AlexNet architecture’s capability to categorize HSR sceneries from remote sensing data is improved using the pre-training method. For the first time, the AlexNet architecture’s easy and comprehensive representation ability can be utilized in HSR remote sensing imaging scene categorization due to the pre-training approach.

(1) f(x)=max(x,0)

### 3.4. Features Classification

**Figure 4:**
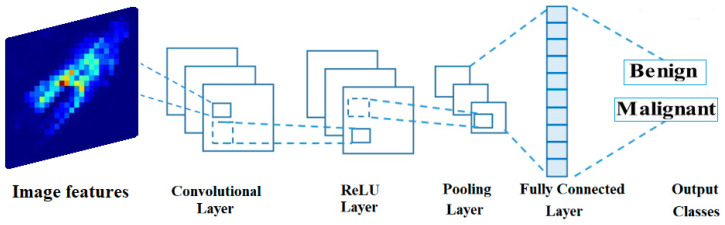
The typical structure of the convolutional neural network used in image classification.

Recently, the most effective neural networks for image processing and classification are convolutional neural networks (CNNs). Feedforward neural network (FFN) models, such as CNNs, allow the signal to propagate in a single direction inside the network without returning to the input node. Since CNN preserves the spatial correlations after filtering the input images, it is one of the best machine-learning (ML) techniques used in medical image analysis. The medical analytic community places a premium on these connections. This section presents a high-level overview of the components that make up CNNs. As may be seen in Figure 4, CNN is made up of several different layers. These are the levels:

#### 3.4.1. Convolutional Layer

Convolution is a procedure that consists of two steps in image analysis. The first step is to enter the pixel values of the features extracted from AlexNet. The second activity is represented by a numeric array called a kernel (or filter). The dot product of the two operations gives the result. The kernel is then moved to the position in the image indicated by the stride length. By iterating the computation until the entire image is covered, a feature map (or activation map) is produced. This map indicates the locations at which the kernel is sufficiently motivated to “see” a feature, such as a straight line, a dot, or a curved edge. For instance, when fed an image of a face, a CNN’s kernels would first identify the image’s underlying low-level features, such as its borders and lines. Low-level features, such as the shape of a person’s ear, eye, or nose, are gathered to produce incrementally better features in the successive layers of a CNN, with the resulting feature maps serving as inputs to the next layer. Convolution relies on sparse connections, weights (parameter) sharing and invariant (equivariant) representation efficient computational machine learning. In contrast to other neural networks, in which all of the neurons in a given layer’s outputs are connected to the inputs of the next layer’s neurons, CNN uses sparse connections, meaning that only a subset of the outputs from each layer is passed along to the next. By gradually learning the important features and drastically reducing the estimated number of weights, the algorithm’s performance improves as the kernel’s covered area per stride (local reception field) diminishes [52]. A CNN can save on memory space by having each kernel’s predefined weights cross over to other parts of the entire image. Unlike in partially connected networks, when weights are used repeatedly between layers, they are only used once in completely linked networks. The quality of the invariant representation improves due to weight sharing, which means that identical translations of the input lead to identical translations of the feature map. The adopted structure of the CNN is shown in Figure 4.

#### 3.4.2. ReLU Layer

This trigger layer makes the input zero if it is less than one. The Rectified Linear Unit (ReLU) layer speeds up training, reduces computational complexity, and aids in avoiding the vanishing gradient problem. The mathematical expression for this is: f(x)=max(0,x). *x* stands for the data coming into the neuron. Parametric ReLU, randomized ReLU, leaky ReLU, tanh, and the sigmoid functions are all examples of additional triggered functions.

#### 3.4.3. Pooling Layer

The pooling layer’s primary purpose is to reduce the image’s dimensions (in horizontal and vertical planes, but not in-depth) and the parameters used to create it. It comes after the convolutional layer but before the ReLU layer. Average and maximum pooling are the two most used methods. The difference between max pooling and average pooling is that the former takes the maximum value of the input within a kernel and discards the others, while the latter takes the average.

#### 3.4.4. Fully Connected Layer

As the last component of the CNN architecture, the fully connected layer ensures that every neuron below it is linked to every neuron in the layer above it. One or more may be used, just as with pooling, ReLU, and convolutional layers, depending on the desired level of feature abstraction. Classification probabilities are computed based on the layer’s output before it (whether pooling, ReLU, or convolutional). To put it another way, the fully connected layer analyzes the most strongly activated features that can assign the image to a certain category. If the features were significantly distinguishable from the preceding layer, the CNN might be beneficial for predicting the presence of cancer cells. The CNN may be trained to discover meaningful structures in previously trained images using the standard neural network training methods of stochastic gradient descent and backpropagation.

#### 3.4.5. Network Hyperparameters

The network structure hyperparameters are listed in Table 3. These parameters determine the structure of the adopted CNN used in feature classification. In addition, the network trained hyperparameters are listed in Table 4. These parameters are trained and optimized using the proposed optimization algorithm. The optimization process results are the best set of parameters that determine the structure of the CNN in addition to the best values of the training hyperparameters used to achieve the best classification accuracy.

### 3.5. The Advanced Al-Biruni Earth Radius Optimization Algorithm

To achieve a better balance between exploitation and exploration, this algorithm partitions the population into subgroups and dynamically adjusts the size of each subgroup. Step one involves creating two groups, one for explorers and one for exploiters. The proportion of the population engaged in exploration is 70%, while that engaged in exploitation is 30%. The exploitation task’s population share is set at 30% of the total population and then gradually increased to 70% over the optimization iterations to increase the fitness values of individuals in each group. However, the initial number of individuals assigned to the exploration group is set at 70%, and via a series of iterations, this number is reduced to 30%. The overall fitness of humans can be vastly enhanced by this method. Furthermore, the elitism technique is used by holding on to the process’s leading answer if no better solution is found; this ensures that the optimization process for the population will converge. Suppose a solution’s fitness does not increase much after three iterations in the BER optimization procedure. In that case, the solution may have reached a local optimum, in which case another exploring individual can be formed using the mutation operation.

For each iteration, the ABER selects the optimal option to implement, guaranteeing a high standard of results. The elitism approach improves the effectiveness of algorithms, but it can lead to early convergence in multimodal functions. The ABER’s mutation process and ensuing search around members of the exploration group provide exceptional exploration capabilities. Due to its robust exploratory capacities, the ABER can delay the onset of convergence. In Algorithm 2, the ABER pseudo-code is displayed. To begin, we feed the ABER some information by specifying the number of iterations, the size of the population, and the mutation rate. The ABER then divides the participants into two groups: the exploration group and the exploitation group. During iterations of the search for the optimal solution, the ABER algorithm dynamically adjusts the size of each group. Each team uses a different method to carry out its duties. With each iteration, the ABER shuffles the order of the solutions to increase diversity and exploration. A solution may belong to the exploration group in one iteration, but it may be part of the exploitation group in the next. Using the ABER’s elitist approach, the leader is less likely to be removed as the process iterates. The steps of the proposed ABER algorithm are presented in Algorithm 2.
**Algorithm 2**: The proposed ABER optimization algorithm1:**Initialize** BER population $P_{i}\left(i=1,2,...,d\right)$, max iterations $Max_{iter}$, population size *d*, fitness function $F_{n}$, initial iteration t=1, intermediates variables *z*, $r_{1}$, $r_{2}$, $r_{3}$.2:**Calculate** fitness function $F_{n}$ for each $P_{i}$3:**Find** best solution as $P^{*}$4:**while** $t\leq Max_{iter}$ **do**5:   **for** each solution in the exploration group **do**6:       **Update** $r=h\frac{cos(x)}{1-cos(x)}$7:       **Calculate** $D=r_{1}\left(P\left(t\right)-1\right)$8:       **Update** $P\left(t+1\right)=P\left(t\right)+D\left(2r_{2}-1\right)$9:   **end for**10: **for** each solution in the exploitation group **do**11:     **Calculate** $D=r_{3}\left(L\left(t\right)-P\left(t\right)\right)$12:     **Update** $P\left(t+1\right)=(r_{1}*P_{1}\left(t\right)+z*r_{2}*\left(P_{2}\left(t\right)-P_{3}\left(t\right)\right)+\left(1-z\right)*r_{3}*\left(P^{*}\left(t\right)-P_{1}\left(t\right)\right)$13:     **Calculate** $k=1+\frac{2*t^{2}}{{Max_{iter}}^{2}}$14:     **Investigate** area around best solution as           $P^{\prime }\left(t+1\right)=r_{1}\left(P^{*}\left(t\right)+k\right)$15:     **Compare** P(t+1) and $P^{\prime }\left(t+1\right)$ to select the best solution $P^{*}\left(t+1\right)$16:     **if** no change occured to the best fitness for last two iterations **then**17:        **Mutate** solution as $P\left(t+1\right)=k+\frac{P_{1}\left(t\right)+P_{2}\left(t\right)+P_{3}\left(t\right)}{e^{zk}}$18:     **end if**19:   **end for**20:   **Update** fitness $F_{n}$ for each position *P*21:**end while**22:**Return** $P^{*}\left(t\right)$

## 4. Experimental Results

In this part, we provide and discuss the results of the proposed architecture for breast cancer classification. Two datasets have been adopted in the conducted experiments, and the achieved results are compared to the other techniques [53,54,55,56,57]. In addition, a cross-validation value of five folds and a training/testing split of 70:30 are applied to improve the achieved accuracy. On the other hand, the proposed optimization approach is compared to different recent approaches, including genetic algorithm (GA) [58], whale optimization algorithm (WOA) [59], particle swarm optimization (PSO) [60], grey wolf optimization (GWO) [61] and the standard Al-Biruni Earth radius (BER) [62]. The parameters of the CNN are optimized using the suggested state-of-the-art BER method. There are many iterations performed to arrive at the final findings, including (i) testing the adopted datasets based on the extracted deep features using other models and (ii) testing the adopted datasets using the extracted deep features and the optimized CNN. All tests are performed on a 16 GB RAM, 8 GB graphics card, MATLAB 2022a-powered desktop computer.

### 4.1. Evaluation Criteria

Table 5 compares the performance metrics used to evaluate the results of the proposed approach. Among these are Negative Predictive Value (NPV), F-score, Precision, Sensitivity, Accuracy, and Specificity. The classification efficiency of the proposed improved CNN is measured using these criteria. The table’s abbreviations for “false negative”, “false positive”, “true negative”, and “true positive” are “FN”, “FP”, “TN”, and “TP”, respectively.

### 4.2. Configuration Parameters

Due to the random initialization of the individuals in the first population, we ran 30 iterations of the optimization algorithms in all the conducted tests. There were 500 iterations in each run. The population is one of the inputs to the algorithm. In this study, that number is 30 individuals. Table 6 details the proposed algorithm’s default settings for its initial parameters.

### 4.3. Feature Extraction Results

The evaluation of the extracted features using transfer learning is presented in Table 7. Starting with accuracy, this table is a commonly used metric that measures the overall correctness of the model’s predictions. In this case, all three models achieved accuracy values greater than 0.81, indicating that they can make correct predictions for most cases. However, it is important to note that accuracy can sometimes be misleading if the dataset is imbalanced, i.e., if one class is much more prevalent. Moving on to sensitivity and specificity, these measures are particularly relevant for binary classification problems such as breast cancer classification. Specificity measures the proportion of true negatives that are correctly identified, while Sensitivity measures the proportion of true positives the model correctly identifies. In this case, the sensitivity values for the models ranged from 0.427 to 0.440, indicating that they can identify true positive cases with comparable performance. The specificity values ranged from 0.925 to 0.949, indicating that the models can correctly identify true negative cases with varying degrees of success. It is important to note that sensitivity and specificity can be affected by the choice of the decision threshold, and different thresholds may result in different performance levels. The Precision and NPV are also relevant evaluation metrics for binary classification problems, as they provide information on the prevalence of false positives and false negatives, respectively. The NPV measures the proportion of positive cases incorrectly classified as negative, whereas the Precision measures the proportion of negative cases incorrectly classified as positive. In this case, the Precision ranged from 0.658 to 0.669, indicating that the models have relatively low rates of false positive predictions. The NPV ranged from 0.846 to 0.889, indicating that the models have somewhat higher rates of false negatives. Finally, the F-score is a measure that combines both precision and recall into a single value. It provides a valuable summary of the model’s overall performance in correctly identifying positive and negative cases. In this case, the F-score values ranged from 0.521 to 0.529, indicating that the models have similar precision and recall, but their ability to balance the two can vary. These evaluation metrics provide a comprehensive view of the performance of the evaluated models for breast cancer classification. By considering multiple metrics, it is possible to gain a more nuanced understanding of the strengths and weaknesses of each model, and to make more informed decisions about which model to use for a particular task. As presented in Table 7, it can be shown that the performance of the AlexNet pre-trained model is superior to the other models for both Dataset-1 and Dataset-2 and, thus, this model is adopted for feature extraction.

### 4.4. Classification Results

Breast cancer classification results using the proposed ABER-CNN compared to the baseline CNN and the optimized CNN using different optimization algorithms are presented in Table 8. The reported results are accuracy scores for five other convolutional neural network (CNN) models: WOA-CNN, GA-CNN, PSO-CNN, GWO-CNN, BER-CNN, and ABER-CNN, that were trained and tested for breast cancer classification. These models were trained using different optimization algorithms, and the reported accuracy scores ranged from 0.914 to 0.962. Among the five evaluated models, the ABER-CNN model achieved the highest accuracy score of 0.962, which suggests that it performed the best in classifying breast cancer.

**Figure 5:**
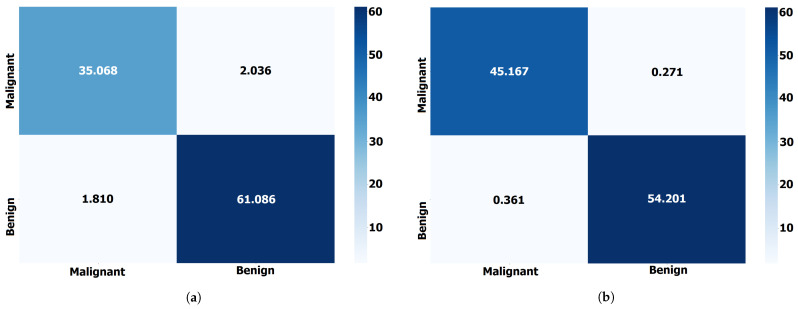
The confusion matrix of the classification results of breast cancer cases using the proposed ABER-CNN applied to the adopted datasets (Dataset-1 and Dataset-2). (**a**) Confusion matrix for Dataset-1. (**b**) Confusion matrix for Dataset-2.

The other models achieved accuracy scores ranging from 0.914 to 0.943. It is important to note that accuracy is only one evaluation metric, and other metrics such as sensitivity, specificity, and F-score may be necessary to evaluate the models’ performance fully. Additionally, further information about the dataset and the specific task would be necessary to fully interpret and contextualize these results. These results suggest that the proposed ABER-CNN model is a promising approach for breast cancer classification, achieving a high accuracy score of 0.962. Similarly, the performance of the proposed approach in terms of Dataset-2 is also presented in Table 8. The results presented in this table confirm the effectiveness and superiority of the proposed approach in breast cancer classification tasks when tested on the adopted datasets. On the other hand, Figure 5 shows the confusion matrix for the results of the proposed ABER-CNN approach applied to Dataset-1 and Dataset-2. From these matrices, it can be noted that the classification of the breast cancer cases is accurate using the proposed approach, which proves its effectiveness in this domain of medical diagnosis.

**Figure 6:**
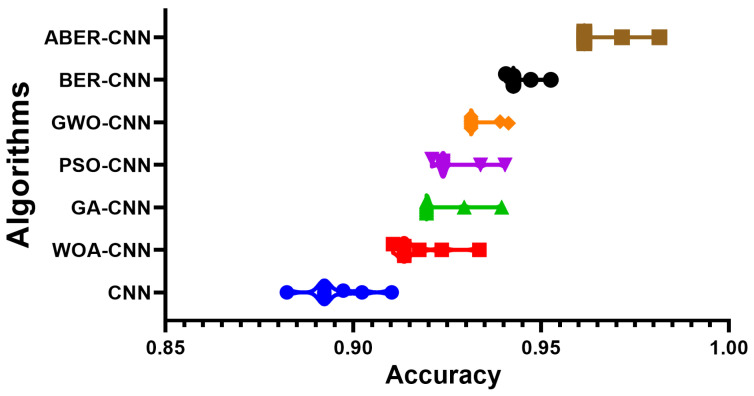
The accuracy of the classification results using the proposed approach compared to other approaches applied to Dataset-1. The colors refer to the corresponding algorithms located on the *y*-axis.

**Figure 7:**
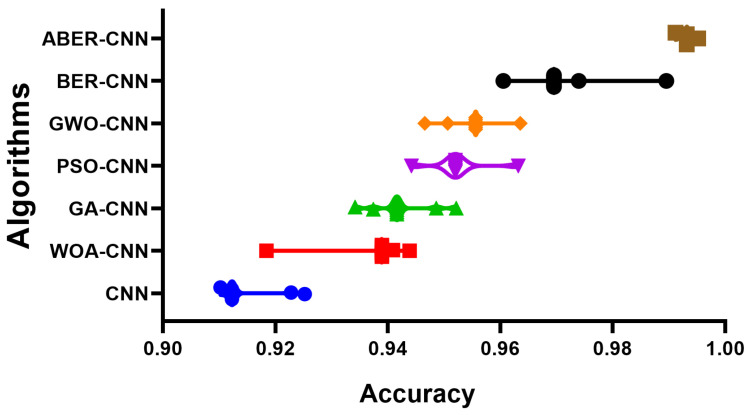
The accuracy of the classification results using the proposed approach compared to other approaches applied to Dataset-2. The colors refer to the corresponding algorithms located on the *y*-axis.

**Figure 8:**
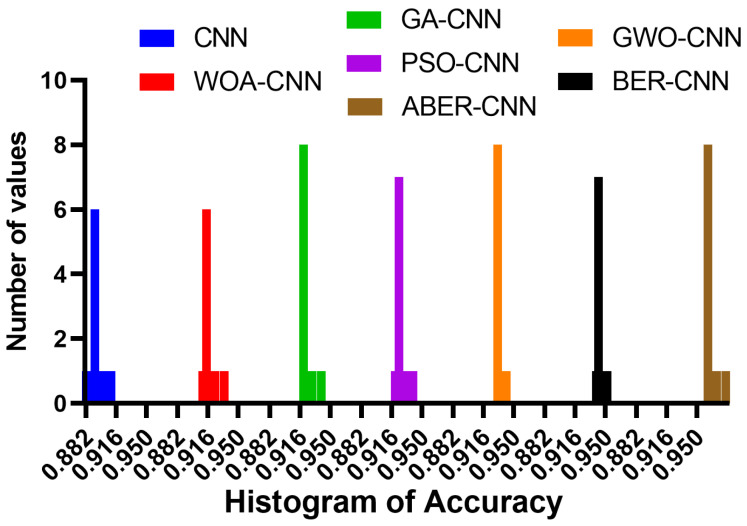
The accuracy histogram of the classification results using the proposed approach compared to other approaches applied to Dataset-1.

**Figure 9:**
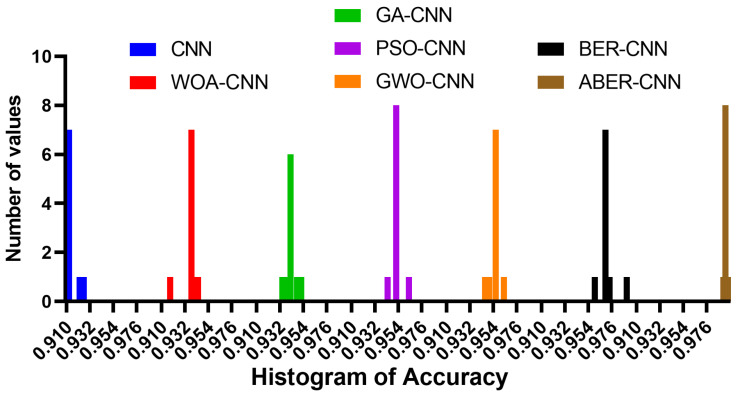
The accuracy histogram of the classification results using the proposed approach compared to other approaches applied to Dataset-2.

The accuracy plot and accuracy histogram plot are valuable tools used to compare the performance of several models in classifying breast cancer cases as shown in Figure 6, Figure 7, Figure 8 and Figure 9 for Dataset-1 and Dataset-2. In this context, the models evaluated include CNN, WOA-CNN, GA-CNN, PSO-CNN, GWO-CNN, BER-CNN, and ABER-CNN, where ABER represents the advanced Al-Biruni Earth radius optimization algorithm, and the proposed approach is ABER-CNN. The accuracy plot visually presents the accuracy scores of each model, allowing for a direct comparison of their performance. It typically displays the accuracy rates on the *y*-axis and the different models on the *x*-axis. This plot enables researchers to assess which model consistently achieves higher accuracy rates in classifying breast cancer cases.

Similarly, the accuracy histogram plot provides a distribution of accuracy scores for each model. It offers a more detailed view of the performance by illustrating the frequency of accuracy scores within specific ranges. This plot allows for comparing the overall accuracy and the accuracy distribution across different models. By analyzing these plots, it becomes evident that the proposed optimized model, ABER-CNN, outperforms the other models in classifying breast cancer cases. Its accuracy scores consistently exceed those of CNN, WOA-CNN, GA-CNN, PSO-CNN, GWO-CNN, and BER-CNN. The superior performance of ABER-CNN suggests that the advanced Al-Biruni Earth radius optimization algorithm effectively enhances the CNN architecture for breast cancer classification. This finding highlights the potential of the ABER-CNN model for more accurate and reliable breast cancer diagnosis, paving the way for improved patient outcomes and healthcare practices in the field. Additional experiment is performed to study the area under the curve (AUC) for the results achieved by the proposed approach when applied to Dataset-1. The results of this experiments are presented in Appendix A.

### 4.5. Statistical Analysis Results

The statistical analysis results are presented in Table 9 for Dataset-1 and Dataset-2. In this table, the results show the performance of different models in terms of various evaluation metrics. The models evaluated include CNN, WOA-CNN, GA-CNN, PSO-CNN, GWO-CNN, BER-CNN, and ABER-CNN. The evaluation metrics reported have the minimum value, 25th percentile, median, 75th percentile, maximum value, range, mean, standard deviation, standard error of the mean, and sum. Looking at the minimum and maximum values, we can see that the ABER-CNN model performed the best, with a maximum value of 0.982 and a minimum value of 0.962. The range of values also varied among the models, with the BER-CNN model having the smallest range of 0.012 and the CNN model having the largest range of 0.028. In terms of the mean and median values, we can see that the ABER-CNN model performed the best, with a mean value of 0.965 and a median value of 0.962. The models’ performances can be compared using the various evaluation metrics provided in the table. The standard deviation values show us that the CNN, WOA-CNN, GA-CNN, PSO-CNN, and ABER-CNN models had similar levels of variability in their results, with standard deviation values ranging from 0.007 to 0.004. The GWO-CNN and BER-CNN models had lower levels of variability with standard deviation values of 0.003. These results suggest that the ABER-CNN model performed the best among the models evaluated.

### 4.6. Analysis-of-Variance (ANOVA) Test Results

The ANOVA table shown in Table 10 displays the findings of a statistical analysis of variance performed on Dataset-1 and Dataset-2. Total, Treatment, and Residual comprise its three sections. The degrees of freedom (DF), mean square (MS), F-ratio (F), and *p*-value for the analysis of variance between treatment groups (models) are displayed in the Treatment section. The treatment has a DF of 6 and MS of 0.00481 (SS: 0.029). There is statistical evidence that the treatment (several models) affects the response variable, as the F-ratio with 6 and 63 degrees of freedom is 131.4, and the *p*-value is less than 0.0001 (evaluation metrics). Unaccounted-for differences between groups of patients are reflected in the Residual term. It has a DF of 63, an MS of 0.00003661, and an SS of 0.002. Residual is omitted because they do not qualify for either the F-ratio or the *p*-value. Since the Total reflects the full range of variability in the data, it displays Total SS, Total DF, and no MS, F-ratio, or *p*-value. The data set has a total of 0.031 SS and 69 DF. In conclusion, the variance table analysis displays the statistical test findings to determine if the intervention (several models) significantly affects the dependent variable. Based on the metrics utilized for comparison, the outcomes highlight a clear performance gap between the various models.

**Figure 10:**
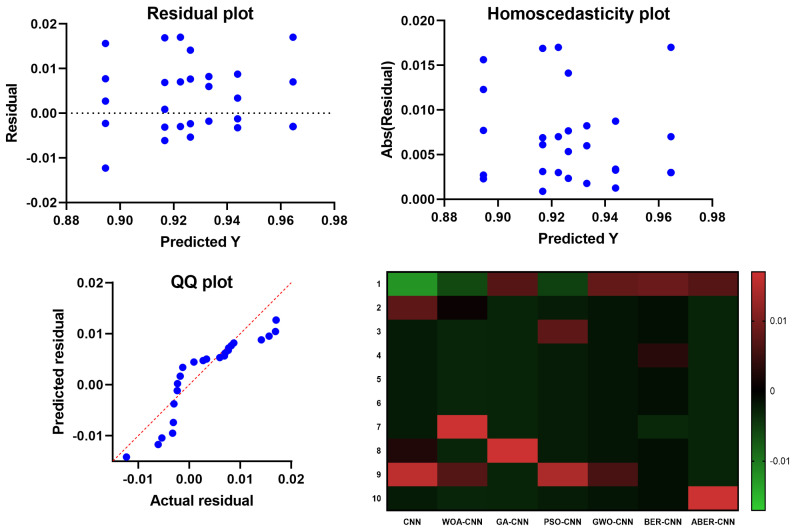
The plots visualizing the results of the ANOVA test based on Dataset-1. The blue dots refer to the samples included in the ANOVA test.

**Figure 11:**
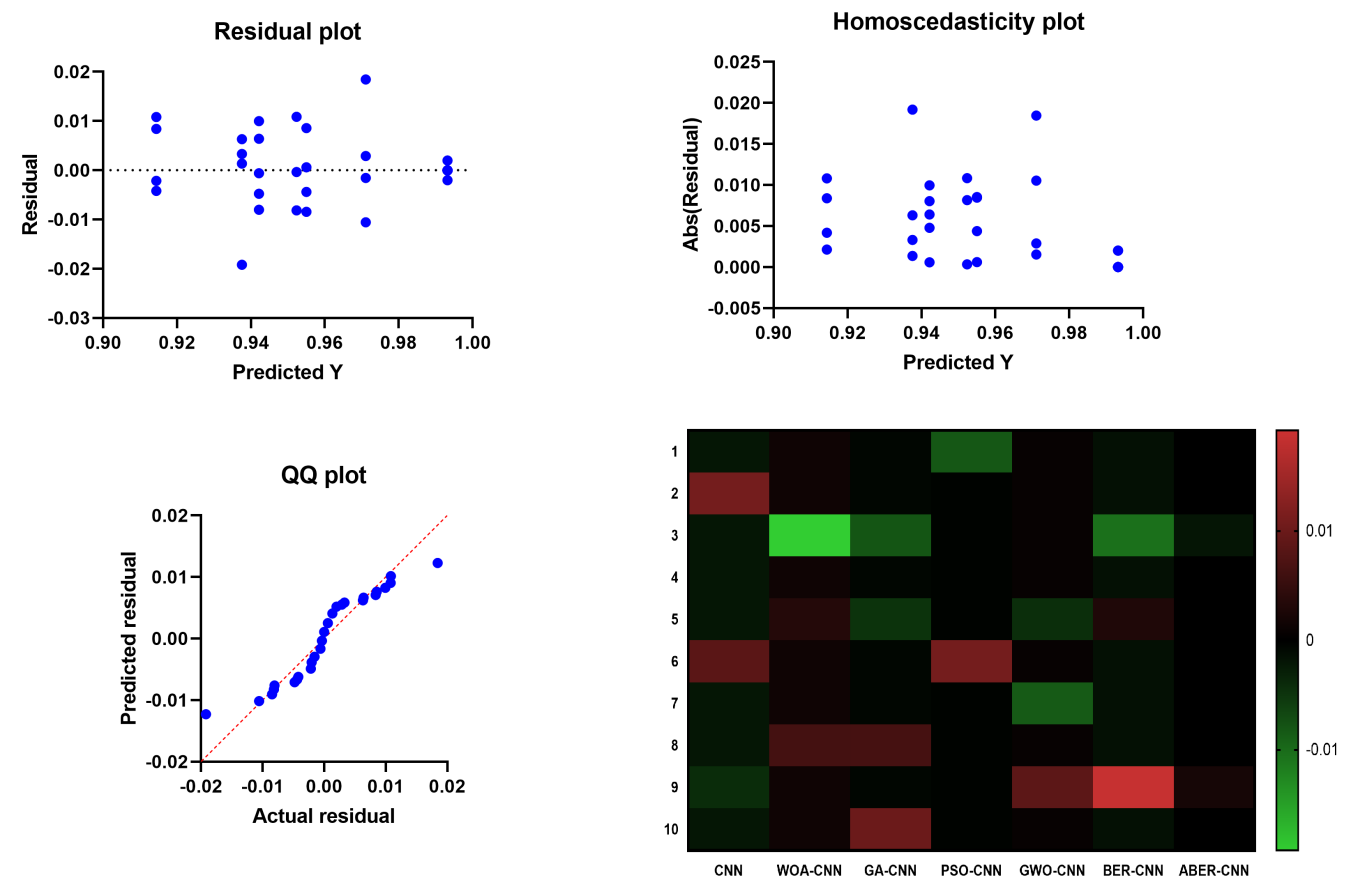
The plots visualizing the results of the ANOVA test based on Dataset-2. The blue dots refer to the samples included in the ANOVA test.

The results of the plots shown in Figure 10 and Figure 11 used to visualize the output of the ANOVA test further validate the effectiveness of the proposed ABER-CNN model in breast cancer classification. Firstly, the QQ plot demonstrates that the residuals of the ABER-CNN model align closely with the expected normal distribution. This indicates that the assumptions of normality are met, enhancing the reliability of the model’s predictions. Additionally, the Homoscedasticity plot reveals a consistent spread of residuals across different independent variable levels, confirming the homoscedasticity assumption. This suggests that the ABER-CNN model performs consistently well across various conditions or groups, further strengthening its robustness in breast cancer classification. The Residual plot showcases minimal patterns or systematic deviations, indicating that the ABER-CNN model effectively captures the underlying linear relationships. The absence of non-linear patterns implies that the model is well-suited for breast cancer classification tasks, as it accurately captures the complexities present in the data.

Furthermore, the Heatmap highlights the significance levels or *p*-values resulting from the ANOVA test. The heatmap reveals that the ABER-CNN model exhibits significantly higher accuracy rates than other models, such as CNN, WOA-CNN, GA-CNN, PSO-CNN, GWO-CNN, and BER-CNN. The color-coded representation indicates the superiority of the ABER-CNN model in classifying breast cancer cases, further supporting its effectiveness and demonstrating its potential for improved patient outcomes and healthcare practices in breast cancer diagnosis.

The results of the QQ plot, Homoscedasticity plot, Residual plot, and Heatmap collectively confirm the effectiveness of the proposed ABER-CNN model in breast cancer classification. These plots provide strong evidence of the model’s accuracy, adherence to assumptions, and robust performance, solidifying its potential as a valuable tool in the early detection and diagnosis of breast cancer.

### 4.7. Wilcoxon Signed-Rank Test Results

The Wilcoxon signed-rank test presented in Table 11 is a non-parametric statistical method for comparing three or more samples with common features. In this context, the test is used to evaluate the relative merits of seven distinct models for a binary classification task: CNN, WOA-CNN, GA-CNN, PSO-CNN, GWO-CNN, BER-CNN, and ABER-CNN. In this test, the median of the observed performance gaps between the models is compared to the theoretical median, which is zero. The findings show that all seven models performed significantly differently from the theoretical median (*p*-value 0.05). Actual median values vary from 0.892 to 0.962, demonstrating various model performances. If we add up all the ranks that represent disparities in absolute value between the observed values and the hypothesized median, we obtain W, the sum of signed ranks. Adding up the ranks of the positive differences yields the total of positive ranks, whereas adding up the ranks of the negative differences yields the sum of negative ranks.

Because the *p*-values are derived from the true probability distribution of the test statistic, the Wilcoxon signed-rank test is considered an exact test. The *p*-values are exactly 0.002, which is a very small probability. The Wilcoxon signed-rank test verifies that there are substantive differences in the effectiveness of the various models. It does not, however, specify how large these disparities are. The deviation numbers reveal the true median values of the performance discrepancies, with ABER-CNN doing better than CNN by a wide margin. One important thing to keep in mind about the Wilcoxon signed-rank test is that it is a one-tailed test, which means that it can only tell you if the models perform considerably better or worse than the theoretical median. It is not a test for directional variations in performance.

## 5. Conclusions and Future Work

In this study, we presented an automated approach for classifying breast cancer cases. Researchers have developed a novel optimization method using the ABER optimization algorithm to improve breast cancer case classification. The proposed system consists of three phases: data augmentation, feature extraction with AlexNet based on transfer learning, and CNN optimization for classification. The proposed approach is evaluated using the two publicly datasets, with an average classification accuracy of 97.95% being attained. Further tests, including ANOVA and Wilcoxon tests and the evaluation of various statistical analysis metrics, are performed to ensure the statistical significance and difference between the proposed approach. The tests validated the suggested methodology’s efficiency and statistical differentiation compared to contemporary approaches. Applying transfer learning and optimized CNN for classification increased classification accuracy when comparing the achieved findings to those of current techniques. The potential limitation of the proposed approach is the complexity of the proposed optimization algorithm, which can be improved by utilizing more flexible nature-inspired algorithms to improve the proposed approach’s overall exploration and exploitation capacities. On the other hand, the future perspectives include evaluating the proposed approach using additional larger datasets and comparing the proposed methodology with more recent approaches.

## Figures and Tables

**Table 1:** Summary of related works.

Ref.	Methodology	Advantage	Disadvantage	Performance
[37]	Modified VGG (MVGG)	Reduce the false positive and false negative rates and the efficiency of mammography analysis is improved	The dataset is not detailed and of small size	94.3%
[38]	Faster R-CNN	High performance in terms of higher sensitivity and lower false positive rate and high efficiently	Could not detect whether the mass lesions are benign or malignant	N/A
[39]	Pretrained InceptionV3 Classifier	High performance in detecting the mass lesions	Long time to detect whether the mass lesions are benign or malignant	96%
[40]	BC-DROID	The ability to classify the images at very low error rates	Complex model	95%
[41]	GAN based data augmentation	Increases samples and assists with class balancing	Low accuracy	75%
[42]	YOLO	Accuracy is high	Needs a large of dataset	95.5%
[43]	CNNI-BCC	Accurate in detecting and classifying breast cancer lesion	Time complexity is high	90.71%
[44]	BDR-CNN-GCN	Effective for data augmentation and accurate detection of malignant breast masses	Time complexity is high	96.10%

**Table 2:** The datasets information along with the number of images before and after data augmentation.

Dataset	Classes	Before Augmentation	After Augmentation
Dataset-1	2 (Benign and Malignant)	1696	6784
Dataset-2	2 (Benign and Malignant)	1356	5424

**Table 3:** Network structure hyperparameters.

Hyperparameter	Abbreviation	Range
Number of Filters 1	Filters_1	[16, 32, 64, 96]
Kernel Size 1	Ksize_1	[3, 4, 5]
Number of Filters 2	Filters_2	[48, 64, 96, 128]
Kernel Size 2	Ksize_2	[3, 4, 5]
Number of Filters 3	Filter_3	[64, 96, 128]
Kernel Size 3	Ksize_3	[3, 4, 5]
Fully connected	full_hidden1	[60, 100, 125]
Activation	activation	[relu, lrelu, elu]

**Table 4:** Network training hyperparameters.

Hyperparameter	Potential Values
Learning rate	[0.001, 0.003, 0.01, 0.03]
learning rates	[0.001, 0.003, 0.01, 0.03]
Batch Size	[0.001, 0.003, 0.01, 0.03]
Dropout	[0.2, 0.3, 0.4, 0.5, 0.6]

**Table 5:** The adopted evaluation metrics.

Metric	Value	
Specificity	$\frac{TN}{TN+FP}$	(2)
Sensitivity	$\frac{TP}{TP+FN}$	(3)
Accuracy	$\frac{TP+TN}{TP+TN+FP+FN}$	(4)
Precision	$\frac{TP}{TP+FP}$	(5)
NPV	$\frac{TN}{TN+FN}$	(6)
F-score	$\frac{TP}{TP+0.5(FP+FN)}$	(7)

**Table 6:** The configuration parameters used for the proposed ABER algorithm.

Parameter	Value
Number of runs	30
Iterations count	500
Population size	30
K (decreases from 2 to 0)	1
Exploration percentage	70%
Mutation probability	0.5
Random variables	[0, 1]

**Table 7:** Evaluating the results of deep networks used in feature extraction.

Dataset-1	Accuracy	Sensitivity	Specificity	Precision	NPV	F-Score
VGG-Net	0.818	0.439	0.925	0.658	0.846	0.526
GoogLeNet	0.835	0.440	0.934	0.663	0.861	0.529
AlexNet	0.867	0.427	0.949	0.669	0.889	0.521
Dataset-2	Accuracy	Sensitivity	Specificity	Precision	NPV	F-Score
VGG-Net	0.843	0.930	0.750	0.800	0.909	0.860
GoogLeNet	0.849	0.933	0.756	0.808	0.912	0.866
AlexNet	0.860	0.938	0.778	0.818	0.921	0.874

**Table 8:** Evaluation of the results achieved by the proposed ABER-CNN compared to the baseline CNN and optimized CNN using different optimization algorithms.

Dataset-1	Accuracy	Sensitivity	Specificity	Precision	NPV	F-Score
CNN	0.892	0.965	0.375	0.917	0.600	0.940
WOA-CNN	0.914	0.970	0.643	0.929	0.818	0.949
GA-CNN	0.920	0.970	0.750	0.929	0.882	0.949
PSO-CNN	0.924	0.970	0.800	0.929	0.909	0.949
GWO-CNN	0.931	0.972	0.833	0.933	0.926	0.952
BER-CNN	0.943	0.968	0.917	0.923	0.965	0.945
ABER-CNN	0.962	0.971	0.945	0.968	0.951	0.969
Dataset-2	Accuracy	Sensitivity	Specificity	Precision	NPV	F-Score
CNN	0.912	0.970	0.833	0.889	0.952	0.928
WOA-CNN	0.939	0.976	0.878	0.930	0.956	0.952
GA-CNN	0.942	0.976	0.887	0.932	0.959	0.953
PSO-CNN	0.952	0.982	0.904	0.943	0.969	0.962
GWO-CNN	0.956	0.984	0.907	0.948	0.970	0.966
BER-CNN	0.970	0.988	0.932	0.968	0.973	0.978
ABER-CNN	0.994	0.993	0.994	0.995	0.992	0.994

**Table 9:** The results of the statistical analysis performing on the results achieved by the proposed approach compared to other approaches.

Dataset-1	CNN	WOA-CNN	GA-CNN	PSO-CNN	GWO-CNN	BER-CNN	ABER-CNN
Number of values	10	10	10	10	10	10	10
Range	0.028	0.023	0.020	0.019	0.010	0.012	0.020
Minimum	0.882	0.911	0.920	0.921	0.931	0.941	0.962
75% Percentile	0.899	0.919	0.922	0.926	0.933	0.944	0.964
25% Percentile	0.892	0.914	0.920	0.924	0.931	0.943	0.962
Median	0.892	0.914	0.920	0.924	0.931	0.943	0.962
Maximum	0.910	0.934	0.940	0.940	0.941	0.953	0.982
Mean	0.895	0.917	0.923	0.926	0.933	0.944	0.965
Std. Error of Mean	0.002	0.002	0.002	0.002	0.001	0.001	0.002
Std. Deviation	0.007	0.007	0.007	0.006	0.004	0.003	0.007
Sum	8.946	9.167	9.225	9.263	9.331	9.439	9.645
Dataset-2	CNN	WOA-CNN	GA-CNN	PSO-CNN	GWO-CNN	BER-CNN	ABER-CNN
Number of values	10	10	10	10	10	10	10
Range	0.015	0.026	0.018	0.019	0.017	0.029	0.004
Minimum	0.910	0.918	0.934	0.944	0.947	0.961	0.991
75% Percentile	0.915	0.939	0.943	0.952	0.956	0.971	0.993
25% Percentile	0.912	0.939	0.941	0.952	0.954	0.970	0.993
Median	0.912	0.939	0.942	0.952	0.956	0.970	0.993
Maximum	0.925	0.944	0.952	0.963	0.964	0.990	0.995
Mean	0.914	0.938	0.942	0.952	0.955	0.971	0.993
Std. Error of Mean	0.002	0.002	0.002	0.001	0.001	0.002	0.000
Std. Deviation	0.005	0.007	0.005	0.005	0.004	0.007	0.001
Sum	9.144	9.376	9.422	9.524	9.55	9.711	9.932

**Table 10:** The ANOVA test outcomes for the comparison models and the proposed approach.

Dataset-1	SS	DF	MS	F (DFn, DFd)	*p*-Value
Treatment	0.029	6	0.005	F (6, 63) = 131.4	*p* < 0.0001
Residual	0.002	63	0.00003661		
Total	0.031	69			
Dataset-2	SS	DF	MS	F (DFn, DFd)	*p*-Value
Treatment	0.038	6	0.006	F (6, 63) = 229.1	*p* < 0.0001
Residual	0.002	63	0.00002753		
Total	0.040	69			

**Table 11:** The Wilcoxon signed-rank test outcomes for the comparison models and the proposed approach.

Dataset-1	CNN	WOA-CNN	GA-CNN	PSO-CNN	GWO-CNN	BER-CNN	ABER-CNN
Actual median	0.892	0.914	0.920	0.924	0.931	0.943	0.962
Theoretical median	0	0	0	0	0	0	0
Number of values	10	10	10	10	10	10	10
Sum of +ve ranks	55	55	55	55	55	55	55
Sum of −ve ranks	0	0	0	0	0	0	0
Sum of signed ranks	55	55	55	55	55	55	55
Discrepancy	0.892	0.914	0.920	0.924	0.931	0.943	0.962
*p*-value	0.002	0.002	0.002	0.002	0.002	0.002	0.002
Dataset-2	CNN	WOA-CNN	GA-CNN	PSO-CNN	GWO-CNN	BER-CNN	ABER-CNN
Actual median	0.9123	0.9389	0.9416	0.952	0.9556	0.9695	0.9932
Theoretical median	0	0	0	0	0	0	0
Number of values	10	10	10	10	10	10	10
Sum of +ve ranks	55	55	55	55	55	55	55
Sum of −ve ranks	0	0	0	0	0	0	0
Sum of signed ranks (W)	55	55	55	55	55	55	55
Discrepancy	0.9123	0.9389	0.9416	0.952	0.9556	0.9695	0.9932
*p*-value	0.002	0.002	0.002	0.002	0.002	0.002	0.002

**Figure A1:**
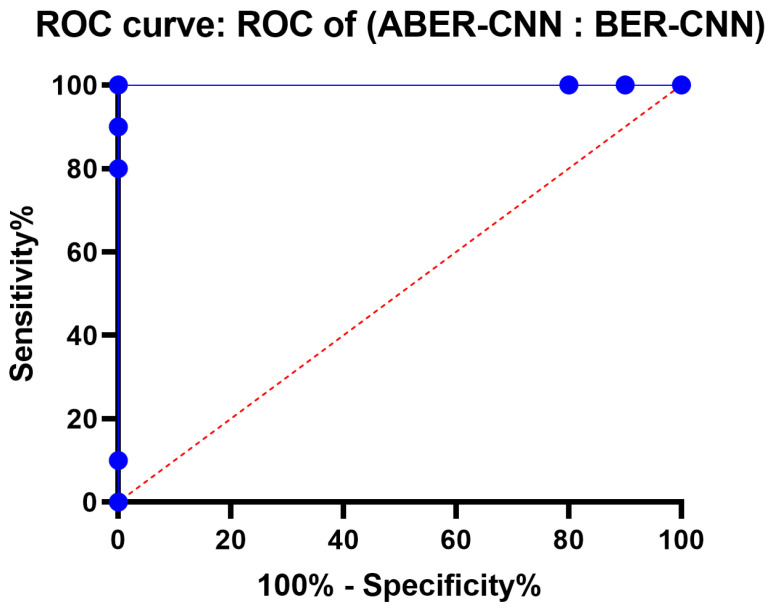
The area under the ROC curve for the results achieved by the proposed approach applied to Dataset-1.

## Data Availability

The datasets employed in this research can be found at the following links. Dataset-1: https://www.kaggle.com/datasets/skooch/ddsm-mammography, Dataset-2: https://dx.doi.org/10.21227/9f0p-qx37.

## Appendix A

The Area under the ROC curve, shown in Figure A1, is a common metric used to evaluate the performance of binary classifiers, such as a CNN model in this case, when applied to Dataset-1. It measures the ability of the model to correctly classify positive and negative cases across all possible classification thresholds. The results show that the AUC score of the model is 1, which indicates perfect discrimination between the two classes (controls and patients). As presented in Table A1, the standard error of the AUC is 0, which means that the estimate of the AUC is very precise. The 95% confidence interval also confirms that the true AUC value is within the range of 1.000 to 1.000, further supporting the conclusion of perfect classification performance.

The *p*-value is 0.0002, less than the commonly used threshold of 0.05, indicating that the observed AUC is significantly different from a random classifier. The data used for the analysis consists of 10 controls (ABER-CNN) and 10 patients (BER-CNN). There were no missing controls or patients in the dataset. These results suggest that the CNN model performs excellently discriminating between controls and patients, with perfect classification performance according to the AUC metric.

**Table A1:** Analysis of the area under the ROC curve.

Metric	Value
Std. Error	0
Area	1
Controls (ABER-CNN)	10
Patients (BER-CNN)	10
Missing Patients	0
Missing Controls	0
*p*-value	0.0002
95% confidence interval	1.000 to 1.000
